# Physical activity as a determinant of successful aging: a narrative review article

**DOI:** 10.1007/s40520-021-02037-0

**Published:** 2021-12-07

**Authors:** Agnieszka Szychowska, Wojciech Drygas

**Affiliations:** Department of Preventive Medicine, Medical University of Lodz, Łódź, Poland

**Keywords:** Healthy aging, Aging, Physical activity, Exercise, Life style, Health behavior

## Abstract

Population of older people in many countries is constantly growing, therefore the subject of successful aging has become important and a priority for public health policy-makers. A person who is successfully aging has low risk of chronic disease and disability, high physical function, good mental health and social engagement in older age. Lifestyle factors, such as diet and exercise, have been identified as determinants of successful aging. The aim of this narrative review is to compile the evidence from big cohort studies on the overall health of older people. Their results indicate that regular physical activity increases the chances of successful aging in older people, but only after reaching a sufficient threshold. Physical activity lowers the risk of many chronic diseases and cognitive decline commonly associated with older age, promotes social engagement and improves self-estimated well-being.

## Introduction

The term “successful aging” (SA) was popularized by Rowe and Kahn in the 1980’s [1]. The Rowe and Kahn model of successful aging focuses on lack of chronic diseases, physical disabilities and risk factors for disease in older age, as well as good mental health, cognitive function and social engagement [2]. Although criticized for the predominantly biomedical approach to aging [3], this model remains one of the most popular in research on this topic. Rowe and Kahn distinguished between “usual” and “successful” aging and recognized the influence of different factors (like diet or exercise) on the process of aging. In the last few decades, many research papers on the impact of exercise on the health and functional status of older people were published and in some of them a term “healthy aging” replaced “successful aging”. World Report on Aging and Health published by World Health Organization describes healthy aging as the “process of developing and maintaining the functional ability that will enable older people to do the things that matter to them” [4]. Because of the lack of consensus among researchers, different definitions of the desirable way of aging are being used. It is also recognized that the definition of successful aging depends on the cultural context of the different communities [5]. However, as the population of older people is growing, and is expected to reach nearly 2.1 billion by 2050 [6], successful/healthy aging remains a priority for public health and health promotion across the world. As a result of the growing population of older people, the official retirement age is being raised in many countries, which means there is a need for workplace health promotion for older workers [7]. However, research shows that poor health can be a risk factor for withdrawing from the labor market before reaching the retirement age [8, 9]. Aging in a successful way means preserving function and independence in older age, which in effect may enable people to be productive even in later life. Avoiding risk factors for chronic diseases and maintaining good health may lead to less absence at work and improved productivity. A lifelong approach to healthy aging is important as investments in health at young age (such as maintaining a healthy and active lifestyle) are profitable throughout life [10]. The aim of this article is to present the current state of knowledge on the impact of physical activity on the process of aging and identify areas that might need to be taken into consideration when designing public health programs and policies. Studies reported in this article have been summarized in tables for better clarity (Tables 1, 2).

**Table 1:** Studies correlating physical activity and successful aging

Study	Type of study	Number of subjects examined	Results
MacArthur study; Berkman et al. [11]	Observational population study	4030	High functioning older people were much more likely to engage in strenuous physical exercise, compared to medium and low functioning groups
Australian Longitudinal Study of Aging (ALSA); Andrews et al. [12]	Observational population study	1403	Lower levels of functioning were associated with lower levels of activity and physical performance
Prognostic Indicator of Cardiovascular and Cerebrovascular Events (PROOF) study; Achour et al. [18]	Observational population study	686	Higher physical activity levels were associated with higher satisfaction with individual health and well-being
POLSENIOR study; Rafał Rowiński [26]	Observational population study	213	Six months of physical activity classes for older people improved their physical function and self-reported quality of life
Almeida et al. [16]	Observational population study	12,201	Participating in at least 150 min of vigorous physical activity per week was associated with improved survival and healthy aging in older men
Part of The 10/66 Dementia Research Group; Daskalopoulou et al. [13]	Observational population study	5000	Older people engaging in physical activity had increased odds of healthy aging
The Blue Mountains Eye Study (BMES); Gopinath et al. [19]	Observational population study	1584	Older adults who engaged in high levels of total physical activity had higher chances of aging successfully 10 years later
English Longitudinal Study of Aging (ELSA); Rogers et al. [21]	Observational population study	8649	Mild physical activity was not associated with better trajectories of frailty progression. Moderate and high levels of physical activity were beneficial on frailty trajectories
WHO SAGE Wave 1 (China); Feng et al. [20]	Observational population study	13,367	150 min/week of vigorous to moderate physical activity was associated with better physical function, better cognitive function, higher quality of life and fewer depressive symptoms
PoliFIT pilot trial; Bernardelli et al. [22]	Observational population study	186	Four months of physical activity classes did not have an effect on the function of older people measured by the time needed to walk 400 m
Tromsø Study; Opdal et al. [14]	Observational population study	12,241	Lack of physical activity was associated with an increased risk of mortality and worse self-reported health compared to physically active subjects
Ageing Trajectories of Health: Longitudinal Opportunities and Synergies (ATHLOS) project; Moreno-Agostino et al. [15]	Observational population study	130,521	Physical activity has a positive impact on the trajectory of aging, supporting healthy aging
Lin et al. [17]	Observational population study (meta-analysis of cohort studies)	189,192	Physical activity was associated with higher chances of successful aging in middle-aged and older people

**Table 2:** Studies concerning the protective mechanism of physical activity towards aging

Study	Type of study	Number of subjects examined	Results
Tan et al. [37]	Case–control study (pilot randomized controlled evaluation)	113	Older adults who were engaging in volunteering showed an increase in their physical activity level
Sofi et al. [30]	Systematic meta-analysis of prospective studies	33,816	Physical activity of all levels showed a protective effect on the occurrence of cognitive decline
ATTICA study; Kollia et al. [27]	Observational population study	853	Higher Healthy Aging Index (which included physical activity) was associated with lower 10-year CVD risk
Lindsay-Smith et al. [35]	Observational population study	28	Group-based social and physical activities programs for older people may improve their well-being and prevent loneliness
Xu et al. [29]	Observational population study	613	Physical activity was associated with a reduced risk of developing metabolic syndrome in the participants with the highest levels of physical activity
de Oliveira et al. [32]	Observational population study	200	There was a correlation between low levels of physical activity and symptoms of anxiety and depression in older people
Steltenpohl et al. [34]	Observational population study	39	Older adults were more likely to prefer to exercise with others, rather than alone
Liu et al. [31]	Meta-analysis of randomized controlled trials	1039	In the elderly with cognitive decline, exercise and nutrition interventions showed a positive effect on global cognitive function
Barnes et al. [33]	Observational population study	6994	Loneliness and social isolation in older people was associated with lower quality of life and higher medical costs

## Physical activity and successful aging

An example of one of the earlier studies on successful aging is the MacArthur study from United States, first reported in 1993 by Berkman et al. Findings of this cross-sectional study of older people (70–79 years of age) point to the fact that subjects characterized as high functioning were much more likely to engage in strenuous physical exercise, compared to subjects from medium and low functioning groups [11]. The MacArthur model of successful aging was also applied in the Australian Longitudinal Study of Aging (ALSA) [12]. Results of this study were similar to the previous one. People aged 70 years and older who were more physically active were also more likely to be classified as high functioning.

In the study by Daskalopoulou et al. four health behaviors were identified as having beneficial impact on successful aging among older people from Cuba, Dominican Republic, Peru, Mexico and Puerto Rico. One of these behaviors was being physically active and results showed that the more active the participants were, the higher odds of healthy aging they had [13].

In the 1970’s Tromsø, the largest city in the Northern Norway, was experiencing high mortality rates due to CVD. To help combat these, the Tromsø Study was initiated, which was later expanded to include other diseases and lifestyle factors. So far, seven health surveys have been performed (between 1974 and 2016) on large cohorts. Physical activity has been found to be associated with reduced overall mortality. Additionally subjects who were achieving an appropriate level of physical activity for their age had good self-reported health for up to 15 years longer than subjects who did not engage in sufficient physical activity. Higher intensities of physical activity (characterized as an activity level that involved sweating/loss of breath) were associated with a stronger positive effect on self-reported health than lighter activities [14].

Using harmonized dataset form eight cohort studies from 26 different countries across the world (i.e. Australia, Denmark, France, Germany, Italy, Japan, Mexico, Poland, Portugal, South Korea, Spain, Sweden, United Kingdom and USA) Moreno-Agostino et al. identified three types of healthy aging trajectories: high stable (displaying a high stable level of health between the starting point of the study and the follow-up), low stable (low level of health in the beginning of the study and almost no change over time) and fast decline (health level similar to the high stable group at baseline with a severe deterioration over time). Findings from this article suggest that people who engaged in some form of physical activity were more likely to exhibit better trajectories of health with the progressing age (the high stable trajectory) compared to people who were classified as physically inactive [15].

Being physically active throughout life is considered a protective factor against many chronic diseases and age-related loss of function. However, studies show that picking up physical activity later in life has similar, but smaller benefits for health. Almeida et al. conducted a study with 10–13 year follow-up, involving 12,201 older men living in Perth, Australia. They found that men who were physically active at baseline (participated in at least 150 min of vigorous physical activity per week) and remained active later in life were more likely to be free of functional and cognitive impairment and depression than those who engaged in less than 150 min of physical activity per week. Moreover, men who were active at baseline but became inactive later in life seemed to have lost the benefits of physical activity on their health. Those, who were classified as physically inactive at baseline, but were classified as physically active at the follow-up, gained the benefits of healthy aging [16]. The conclusion from this study is that it is never too late to implement physical activity in everyday life. However, a meta-analysis by Lin et al. shows that the protective effect of physical activity decreased with age in middle-aged and older adults. Being active as a younger person was beneficial to successful aging later in life, but this effect decreased by approximately 3% annually [17].

Satisfaction with life is an important indicator of successful aging. In the PROOF study (France) Achour et al. found a correlation between the level of physical activity in people over 65 years old and their self-estimated health status and well-being. People with higher physical activity levels were more likely to be satisfied with their health and well-being [18].

Gopinath et al. found that in a population of Australian adults, those engaging in higher levels of physical activity (over 5000 MET min/week according to the International Physical Activity Questionnaire) were twofold more likely to age successfully over 10 years, than those who engaged in less physical activity (below 1000 MET min/week) [19]. Feng et al. found that among older Chinese people, a WHO-recommended amount of at least 150 min/week of vigorous to moderate physical activity was associated with better physical function, better cognitive function, higher quality of life and fewer depressive symptoms. These health outcomes are associated with key elements of successful/healthy aging [20].

When considering the impact of physical activity on the health of older people, an important question is often posed: what is the minimal dose of exercise needed to observe a positive effect? Rogers et al. conducted a study using data from the English Longitudinal Study of Aging (ELSA) on the trajectories of frailty and how physical activity may affect them. Because frailty prevents older people from being independent and significantly lowers their quality of life, it is an important factor preventing successful and healthy aging. They found that mild physical activity, when compared to sedentary behavior, did not show association with improved frailty trajectories. Only moderate and vigorous physical activity were beneficial, compared to being sedentary [21].

The PoliFIT pilot trial from Milan, Italy, involved people 70 years of age and over in an intervention of physical activity classes, carried out weekly, for 4 months. At the end of the 4 months, participants did not show statistically significant improvements in function measured by the 400 m walk test. However, a vast majority of them reported satisfaction with the classes and claimed it helped them in carrying out activities of the daily life. It is possible that the frequency of classes was too low and the 4-month period too short to have a significant impact on the measure of function. These results can be useful for policy-makers when designing physical activity programmes for the elderly [22]. An important factor that has to be considered when developing physical activity recommendations for older people is the impact that the ambient temperature has on the ability to exercise. One consequence of climate change is rising temperatures in more and more places in the world, which means the ability to exercise outside (which is generally more accessible for older people) will become limited. Taking this into consideration, recommendations should include avoiding physical activities (such as walking, jogging, and cycling) outside during the hottest hours of the day, possibly planning such activities for the mornings or evenings. Proper rehydration, using spaces with air-conditioning and pools as well as regular breaks are also important, since older people tend to have diminished ability to thermoregulate, often have more comorbidities and a higher risk of heat-related illnesses [23]. Another way of preparing older people for heat waves is a heat acclimation exercise protocol, shown to improve physiological response to high temperatures. Post-exercise hot water immersion has also shown promising results in acclimating the elderly to the heat and improving responses to heat stress [24].

POLSENIOR research project, a study on health and quality of life of older people in Poland, is an example of a multidisciplinary approach to research on aging. As part of this study, a research project was conducted in 2009, which involved a 6-month physical activity classes program for people 65 years and older. Results showed that there were statistically significant differences in results of the FFFT (Fullerton’s functional fitness test, also known as senior fitness test [25]) between baseline and at the end of the program, meaning that there was an improvement in participants physical function. An improvement was also observed in self-reported quality of life of participants [26].

## Possible mechanisms

What is the mechanism in which PA (physical activity) influences the likelihood of SA (successful aging)? It has been proven that physical activity lowers the risk of many chronic diseases, including CVD [27], cancer [28], obesity and metabolic syndrome [29]. Since lack of chronic disease is one of the main factors in successful aging, it is easy to notice the link between PA and SA.

Another indicator of successful aging is good cognitive function. Sofi et al. performed a systematic meta-analysis of prospective studies investigating the effect of physical activity on the risk of cognitive decline [30]. Findings of this meta-analysis suggest a strong protective effect of all levels of physical activity against cognitive decline in nondemented adults. Analyzing six randomized controlled trials, Liu et al. also found that nutrition combined exercise interventions can have a positive effect on global cognitive function in the elderly with cognitive decline [31]. Physical activity has also been shown to improve anxiety and depression in older people, thus improving their quality of life [32]. Good mental health plays an important role in successful aging.

Loneliness and social isolation in older age have a negative effect on the quality of life. Increased likelihood of depression and greater medical costs are also connected to these constructs [33]. Therefore, social engagement is often included in definitions of successful and healthy aging. Research has found that older adults prefer to exercise with others rather than alone [34]. Group-based exercise programs for older people can provide an opportunity to prevent/reduce loneliness and social isolation [35]. It can also improve levels of social connectedness, apart from other benefits of physical activity in the elderly [36]. Moreover, engaging in social activities like volunteering has been found to be associated with increased level of physical activity [37].

## Conclusions

Physical activity is associated with increased chances of successful aging. Preventing loss of physical and cognitive function and improving mental health and social engagement are the benefits of physical activity that improve chances of aging successfully and healthily. Promoting lifestyle that increases chances of successful aging should be among the priorities of public health policy-makers, as the population of older people is constantly growing. Intensity and frequency of PA should be adjusted to the possibilities of older people, remembering that there is a threshold of minimal physical activity level that needs to be met in order to gain the benefits for health and function.

## Data Availability

Not applicable.

## References

[1] Rowe JW, Kahn RL (1987). Human aging: usual and successful. Science.

[2] Rowe JW, Kahn RL (1997). Successful aging. Gerontologist.

[3] Katz S, Calasanti T (2015). Critical perspectives on successful aging: does it “appeal more than it illuminates”?. Gerontologist.

[4] World Health Organization (2015) World report on ageing and health

[5] Fatemeh E (2020). The concept of successful aging: a review article. Curr Aging Sci.

[6] United Nations, Department of Economic and Social Affairs, Population Division (2017) World population ageing 2017 (ST/ESA/SER.A/408)

[7] Magnavita N (2018). Health promotion for the aging workforce in Poland. Int J Occup Med Environ Health.

[8] Kouwenhoven-Pasmooij TA (2016). Cardiovascular disease, diabetes and early exit from paid employment in Europe; the impact of work-related factors. Int J Cardiol.

[9] Piłat A (2019). Challenges for the labor market: 2 complementary approaches to premature cessation of occupational activity. Int J Occup Med Environ Health.

[10] Nimrod G, Ben-Shem I (2015). Successful aging as a lifelong process. Educ Gerontol.

[11] Berkman LF (1993). High, usual and impaired functioning in community-dwelling older men and women: findings from the MacArthur Foundation Research Network on Successful Aging. J Clin Epidemiol.

[12] Andrews G, Clark M, Luszcz M (2002). Successful aging in the Australian longitudinal study of aging: applying the MacArthur model cross-nationally. J Soc Issues.

[13] Daskalopoulou C (2018). Associations of lifestyle behaviour and healthy ageing in five Latin American and the Caribbean countries-a 10/66 population-based cohort study. Nutrients.

[14] Opdal IM (2020). A prospective study on the effect of self-reported health and leisure time physical activity on mortality among an ageing population: results from the Tromsø study. BMC Public Health.

[15] Moreno-Agostino D (2020). The impact of physical activity on healthy ageing trajectories: evidence from eight cohort studies. Int J Behav Nutr Phys Act.

[16] Almeida OP (2014). 150 minutes of vigorous physical activity per week predicts survival and successful ageing: a population-based 11-year longitudinal study of 12 201 older Australian men. Br J Sports Med.

[17] Lin Y-H (2020). Physical activity and successful aging among middle-aged and older adults: a systematic review and meta-analysis of cohort studies. Aging.

[18] Achour EC (2011). Level of physical activity at the age of 65 predicts successful aging seven years later: the PROOF study. Rejuvenation Res.

[19] Gopinath B (2018). Physical activity as a determinant of successful aging over ten years. Sci Rep.

[20] Feng Z, Cramm JM, Nieboer AP (2019). A healthy diet and physical activity are important to promote healthy ageing among older Chinese people. J Int Med Res.

[21] Rogers NT (2017). Physical activity and trajectories of frailty among older adults: evidence from the English Longitudinal Study of Ageing. PLoS One.

[22] Bernardelli G (2019). Adapted physical activity to promote active and healthy ageing: the PoliFIT pilot randomized waiting list-controlled trial. Aging Clin Exp Res.

[23] See L (2021). Considerations in planning physical activity for older adults in hot climates: a narrative review. Int J Environ Res Public Health.

[24] Waldock KAM (2021). Exercise heat acclimation and post-exercise hot water immersion improve resting and exercise responses to heat stress in the elderly. J Sci Med Sport.

[25] Rikli RE, Jones CJ (1999). Functional fitness normative scores for community-residing older adults, ages 60–94. J Aging Phys Act.

[26] Rafał Rowiński AD (2012) Aktywność fizyczna Polaków w wieku podeszłym. Aspekty medyczne, psychologiczne, socjologiczne i ekonomiczne starzenia się ludzi w Polsce

[27] Kollia N (2018). Determinants of healthy ageing and its relation to 10-year cardiovascular disease incidence: the ATTICA study. Cent Eur J Public Health.

[28] Patel AV (2019). American College of Sports medicine roundtable report on physical activity, sedentary behavior, and cancer prevention and control. Med Sci Sports Exerc.

[29] Xu F (2019). The association between physical activity and metabolic syndrome in older adults with obesity. J Frailty Aging.

[30] Sofi F (2011). Physical activity and risk of cognitive decline: a meta-analysis of prospective studies. J Intern Med.

[31] Liu T (2021). Nutrition and exercise interventions could ameliorate age-related cognitive decline: a meta-analysis of randomized controlled trials. Aging Clin Exp Res.

[32] de Oliveira L (2019). The effects of physical activity on anxiety, depression, and quality of life in elderly people living in the community. Trends Psychiatry Psychother.

[33] Barnes TL et al (2021) Cumulative effect of loneliness and social isolation on health outcomes among older adults. Aging Ment Health 1–8. 10.1080/13607863.2021.1940096 10.1080/13607863.2021.1940096 34215167

[34] Steltenpohl CN (2019). Me time, or we time? Age differences in motivation for exercise. Gerontologist.

[35] Lindsay-Smith G (2018). A mixed methods case study exploring the impact of membership of a multi-activity, multicentre community group on social wellbeing of older adults. BMC Geriatr.

[36] Sebastião E, Mirda D (2021). Group-based physical activity as a means to reduce social isolation and loneliness among older adults. Aging Clin Exp Res.

[37] Tan EJ (2006). Volunteering: a physical activity intervention for older adults–the Experience Corps program in Baltimore. J Urban Health.

